# Comparison of Tensile Strengths of Synthetic and Natural Absorbable Sutures in Minor Oral Surgeries: A Randomized Clinical Trial -

**DOI:** 10.31661/gmj.v13iSP1.3621

**Published:** 2024-12-31

**Authors:** Leila Golpasand Hagh, Mahmood Beiki Ghasemi, Erfan Safikhani, Navid Dorestan

**Affiliations:** ^1^ Department of Periodontics, School of Dentistry, Ahvaz Jundishapur University of Medical Sciences, Ahvaz, Iran

**Keywords:** Chromic Catgut, Polyglycolic Acid, Tensile Strength, Oral Surgery, Sut

## Abstract

**Background:**

Proper suture selection is important in oral surgery for uncomplicated healing. This study was conducted to compare the tensile strength of two absorbable natural and artificial sutures in minor oral surgeries.

**Materials and Methods:**

In this randomized clinical trial study, two types of absorbable sutures, 0-4 Chromic catgut and Polyglycolic Acid (PGA) were randomly used in minor oral surgery in 16 systemically healthy patients. Tensile strength tests were performed for one part of the suture thread before suturing along with sutures removed on days 7 and 10 post-surgery (24 suture threads for each group). Suture parts were tested by a Universal Testing Machine for tensile strength. The results of this study were analyzed using the Kolmograph-Smirnov test, repeated measures analysis of variance, and independent t-test.

**Results:**

The mean tensile strength of chromic catgut suture on day 0 was )10.40±1.61 N), 7th day (8.76±1.66 N), and 10th day )6.45±1.14 N). The mean tensile strength of the PGA suture thread on day 0 was (16.82±2.94 N), 7th (14.56±2.66 N), and 10th day (11.50±2.15 N). The mean tensile strength of the PGA suture was significantly higher at baseline, 7 and 10 days after surgery compared to the absorbable chromic catgut suture (P0.001). In chromic catgut sutures and PGA sutures, the mean tensile strength on day 10 was significantly lower than on days 7 (P0.05) and 0 days (P0.001), and the mean tensile strength on day 7 was significantly lower than on day 0 (P0.05).

**Conclusion:**

The results indicate that PGA sutures maintain significantly higher tensile strength compared to chromic catgut sutures throughout the post-surgical period. This suggests that PGA sutures may be more suitable for minor oral surgeries where higher tensile strength is required for optimal wound healing. Surgeons should consider the specific needs of the surgical site and the duration of healing when selecting the appropriate suture material.

## Introduction

**Figure-1:**
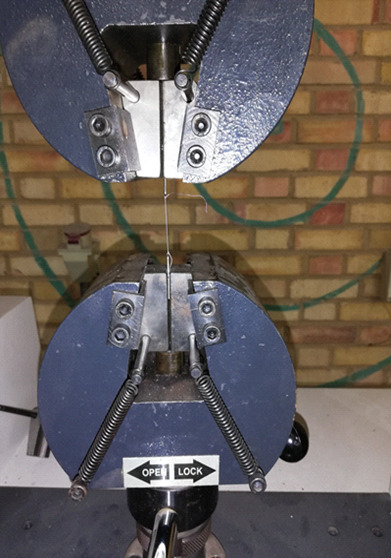
Figure 1. Tensile strength test of two types of chromic catgut and PGA by Universal Testing Machine

Suture thread is the most common material used to close the wound’s edges, control bleeding, and assist wound healing [1][2][3]. Closure of wound edges as water-tight is very crucial in some surgeries, such that it would not allow entrance of saliva to the soft tissue. If the wound undergoes dehiscence, saliva can enter the soft tissue, causing serious problems in the tissue healing process, including fistula formation and wound infection. Since some oral surgeries do not allow the surgeon to remove the sutures, most surgeons use absorbable sutures to close the edges of the wound in these cases. The ideal absorbable suture should be such that it retains its strength until the wound heals and then absorbs quickly so as not to cause inflammatory reactions [4][5].

Absorbable sutures commonly used in the oral cavity include chromic catgut, poliglecaprone, polyglactin, and polyglycolic acid [6]. Absorbable artificial sutures are destroyed by hydrolysis, and naturally absorbable collagen-based sutures are broken down by proteolytic enzymes released by inflammatory cells during wound healing [7]. Absorbable sutures in the oral cavity must withstand mechanical forces from chewing and speaking, as well as biochemical factors like pH changes, proteolytic enzymes, and vascularization, making high tensile strength and minimal tension crucial for optimal wound healing, often necessitating dietary adjustments post-surgery [8]. Previous laboratory studies have shown an association between decreasing suture thread strength and its absorption with various body fluids including synovial fluid, urine, bile, and stomach contents. In one study, it was observed that all types of sutures (whether natural or artificial, monofilament or multifilament) are more degradable in the presence of saliva [8]. One of the important properties of suture that significantly affects its persistence in the oral cavity is the tensile strength of the suture. The term tensile strength refers to the ability of a material to withstand the tensile force before it breaks or tears. Tensile strength is a key feature in sutures, such that if the suture loses this property faster than the wound healing rate, it can cause wound opening, thereby increasing the probability of complications and secondary infection [9]. It has been demonstrated that sutures do not gradually lose their strength over time in a linear manner, but instead experience a decrease in strength in the form of a curve. This reduction can cause the suture to rupture before it is removed [10]. Clinically, inflammation of the surgical flaps during wound healing may cause the edges of the wound to stretch and thus the edges of the flap to open. Sutures with insufficient tensile strength during the healing phase can lead to poor adaptation of the wound edges and hematoma. Most of the studies to measure the tensile strength of suture threads have been done in the laboratory and environments outside of saliva and the biological environment of the mouth, so we decided to investigate the tensile strength of two common types of absorbable suture threads (chromic catgut and polyglycolic acid) after minor oral surgery.

## Materials and Methods

This randomized clinical trial study was performed on 16 patients referring to the Periodontics Department of Jondishapour faculty of dentistry who required minor oral surgery from January to November 2020. The patients were in good overall health, aged between 18 and 55 years, and showed no signs of infection in the surgical area. Before the start of the study, the objectives were described to the patients and informed consent was obtained from them to participate in the study (Ethics Code: IR.AJUMS.REC.1399.172). This study was registered on the Iranian Clinical Trial website with the code IRCT: IRCT20200815048413N1.

A previous study reported a mean tensile strength of 392.276 MPa with a standard deviation of 50 MPa for Chromic catgut monofilament and 1070.292 MPa with a standard deviation of 70 MPa for synthetic braided violet-coated polyglycolide (PGA) sutures [11]. With a power of 80% and a significance level of 0.05, a sample size of 8 patients per group was required. This calculation was performed using G*Power software, version 3.1.9.2. Therefore, 16 patients were included in the study, with 8 patients in each group.

Patients who did not go for suture extraction on days 7 and 10 after surgery or who did not have suture threads in their mouth at the time of the study were excluded from the study. These patients were randomly divided into two groups of 8 patients. In this study, two types of absorbable 4-zero sutures were used for both groups. In one group, Chromic catgut absorbable suture (SUPA CHROMIC, SUPA, Iran) and polyglycolic acid suture (SUPABON, SUPA, Iran) were used to suture the wound edges. Surgeons’ knots were used to reduce the possibility of opening the suture knot. One part of each suture was separated before suturing to perform a tensile strength test to determine the tensile strength of the base. This part of the suture along with the sutures that were removed on days 7 and 10 after surgery were evaluated for tensile strength by Universal Testing Machine (STM 50, SANTAM Eng. Design. Co, IRAN) (Figure-1) (24 sutures for each group and a total of 48 sutures).

The suture thread was tied to two metal clamps to perform the tensile strength test, and if it was not possible to knot, it was fixed to the metal clamp with glue. These two clamps were then placed in the two jaws of the Universal testing machine. The cross-head speed at the time of the test was 5 mm/min. After the surgery, the patients were given the necessary orders and they were emphasized to use chlorhexidine mouthwash regularly twice per day for 1 minute over these 10 days. Also, they should have chewed their food by the opposite side of the mouth. Also, all the steps were performed on both groups by the same periodontics resident.

The results of this study were analyzed using SPSS v. 16.0 (SPSS Inc., Chicago, IL, USA) and Kolmogorov-Smirnov tests, repeated measures analysis of variance, and independent t-tests were used for statistical comparisons. Values less than 0.05 were considered as a significant level.

## Results

**Figure-2:**
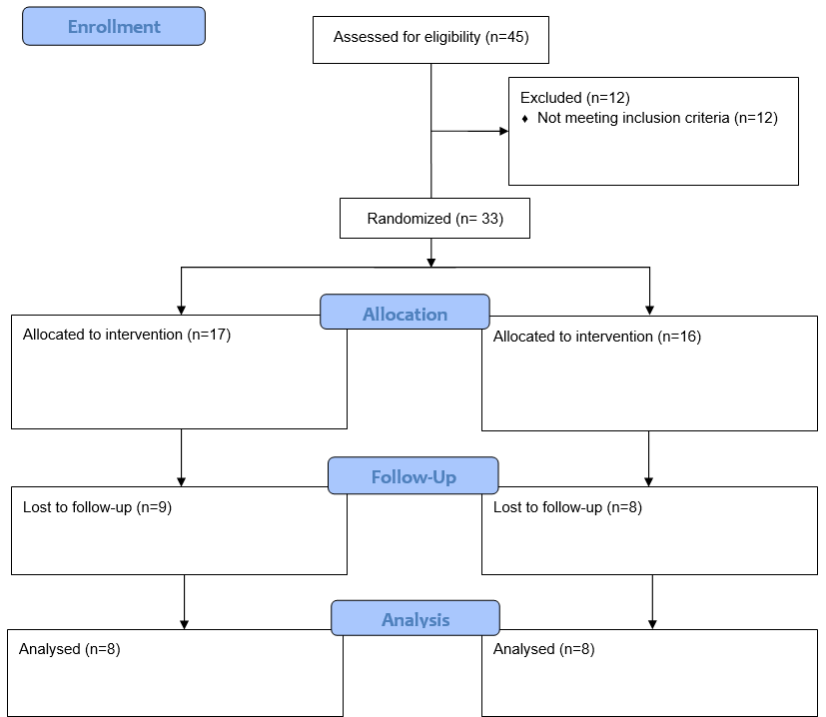
Figure 2. CONSORT flow diagram of the study

**Table:** Table1. Determination and Comparison of Tensile Strength of Chromic Catgut and Polyglycolic Acid Absorbable Suture Thread before Surgery, on Day 7, and, on Day 10 after Surgery

**Evaluation Day**	**Chromic Catgut (n=8)**	**Polyglycolic Acid (n=8)**	**P value**
Day 0 (Beginning of Study)	10.40 ± 1.61	16.82 ± 2.94	<0.001**
Day 7	8.76 ± 1.66	14.56 ± 2.66	<0.001**
Day 10	6.45 ± 1.14	11.50 ± 2.15	<0.001**
P value	<0.001*	<0.001*	

^*^: Analysis of variance with repeated sizes, ^**^: independent t-test.

In the current study, several patients did not return to remove stitches due to the outbreak of the Coronavirus pandemic, and in two patients, there was no chromic catgut suture in the mouth on the 10th day after surgery, these patients were excluded from the study (Figure-2).

The study included 16 patients, with an equal distribution of males and females (4 males and 4 females) in each group. The mean age of the patients was 36.5 years, with a standard deviation of 7.8 years. No significant differences were observed in the age or gender distribution between the polyglycolic acid (PGA) and chromic catgut groups (P>0.05).

The descriptive statistics in comparison of the tensile strength of the absorbable suture threads (PGA and Chromic catgut) were evaluated at different times, and presented in Table-1. According to the Kolmogorov-Smirnov test, tensile strength distribution in the polyglycolic acid and chromic catgut groups was normal before surgery, on days 7 and 10 after surgery (P>0.05).

The Chromic Gut 4-0 suture thread showed 74.23% of its tensile strength on day 7 post-surgery. This level on day 10 post-surgery reached 62.01. The PGA 4-0 on days 7 and 10 post-surgery demonstrated 86.66% and 68.37% of its tensile strength respectively.

The mean tensile strength of the PGA suture thread at baseline as well as days 7 and 10 post-surgery was significantly higher than that of the Chromic Gut absorbable suture thread (P<0.001).

Both suture threads showed their maximum mean tensile strength at the baseline followed by day 7 and then day 10 (Table-1). In the Chromic Gut suture thread, the mean tensile strength was significantly lower on day 10 post-surgery compared to day 7 post-surgery (P=0.006) and day 0 (P<0.001). The mean tensile strength was significantly lower on day 7 compared to day 0 (P=0.004). Regarding the PGA, the mean tensile strength was significantly lower on day 10 compared to days 7 and 0 (P<0.001). Finally, the mean tensile strength was significantly lower on day 7 compared to day 0 (P=0.031).

## Discussion

This study aimed to compare the tensile strength of two absorbable sutures (chromic catgut versus polyglycolic acid) in minor oral surgeries. These two sutures were selected based on their repeated use in different oral surgery interventions.

In this study, the mean tensile strength was significantly decreased in both the absorbable chromic gut and polyglycolic acid sutures from the beginning of the study from the seventh day to the tenth day. This finding is slightly less than the amount provided by the manufacturer, which is 14 to 21 days for chromic gut suture thread and up to 28 days for PGA suture. Alternatively, this finding could be due to the oral environment, which contains different enzymes and pH [12].

This finding was similar to the study of Taysi et al. (2021) in which the tensile strength of polyglycolic acid absorbable suture threads decreased after 14 days [11]. Khiste et al. (2013) reported that the 4-zero PGA suture retains its tensile strength until the seventh day [13]. In an in vivo study conducted by Shaw et al. (1996) on oral tissues, the PGA suture had a shelf life of 15 days [14]. Low-cycle tensile fatigue test showed that the fatigue strength of PGA suture was significantly reduced by cyclic loading and degradation in oral tissues after a period of five to seven days [15].

In a systematic review conducted by Faris et al. (2022), the researchers reported higher tensile strength for silk and nylon compared to Catgut and PGA [2]. Kim et al. (2007) evaluated the tensile strength of the chromic gut suture thread that was immersed in saline solution for 14 days. These researchers reported that the tensile strength of the chromic gut suture thread decreased significantly after this time [10].

In a study, Mathew et al. (2018) investigated the tensile strength of absorbable and non-absorbable suture threads, which were kept in the same oral environment. The PGA suture showed the highest tensile strength among the three types of absorbable sutures (Catgut, PGA, and Polydioxanone) [3]. This finding is consistent with our study.

Briddell et al. (2017) reported that the breakdown of the chromic suture thread began three days after immersion in artificial saliva [8]. In an in vivo study conducted by Fomete et al. (2013) on oral tissues, the chromic suture thread remained in the mouth for 5 to 16 days. In our study, it was also observed that in 2 patients, the chromic gut thread was not present in the mouth on day 10 [12].

However, the ideal time for the degradation of sutures in oral tissues has not been clearly defined. Nevertheless, biologically, the suture must act as long as the edges of the wound develop sufficient tensile strength to stand on its own. Beyond this time, the suture acts merely as a foreign object and may impair the healing potential. Thus, being aware of the timing of events in the healing of oral tissues is very important. This is because, during the acute inflammatory phase of wound healing, the tissues do not acquire significant tensile strength, and rely solely on the closure material to hold the edges of the wound close to each other [12].

The results of this study indicated that the mean tensile strength of polyglycolic acid suture was significantly higher at the beginning of the study, 7 and 10 days after surgery, compared to the absorbable chromic gut. This finding is in line with the findings of studies in which braided sutures have higher tensile strength than single-stranded sutures, and braided sutures resist stretching more before tearing [11][16]. Taysi et al. (2021) evaluated the tensile strength of polytetrafluoroethylene, polypropylene, polyester, silk, polyglactin 910, polyglycolic acid, poliglecaprone 25, and polydioxanone sutures in oral surgeries as in vitro. They found that the tensile strength of polytetrafluoroethylene suture thread during days 3, 7, and 14, remained almost constant, and the rest of the sutures showed a significant decrease in tensile strength.

Monofilament suture threads show less resistance to tissue passage and are less likely to harbor organisms. On the other hand, they must be used carefully, as they can weaken or tear when crushed by a needle holder or another instrument. Monofilament sutures are used in vascular and microvascular surgery. When several strands are woven together, and multifilament suture is produced, more tensile strength, flexibility, and pliability are created [16]. This can be one of the reasons for the higher tensile strength of braided suture thread PGA compared to chromic gut monofilament suture thread.

On the other hand, it should be noted that natural absorbable sutures absorbed by proteolysis cause an inflammatory response, while artificial absorbable sutures absorbed by hydrolysis produce minimal inflammatory reactions. Assuming the same technique, texture, and other factors, the tissue response to all sutures in the first 5 days is relatively the same, and after, the tissue response depends mostly on the type of suture material [12].

In the present study, on the 10th day after surgery, two chromic gut sutures were no longer present in the mouths of two patients. These patients were excluded from the study. The reasons for this could be the following:1- Tensile strength reaching zero. 2- Opening the knot, which is especially common in monofilament suture threads (surgeons’ knots were used to reduce this problem). 3. Tearing of the edges of the wound and the removal of the suture thread (to reduce this problem, the insertion point of the suture needle was considered at least 3 to 5 mm from the edge of the wound). 4. Consuming foods with a different pH and eating the patient from the surgical side. 5. Intense muscle tension in the surgical area. 6. Applying force to the suture through the tongue. It is recommended that in future studies, to prevent the absence of suture thread during the post-surgery period, the following measures should be taken: increase the number of knots, avoid very alkaline foods, inform patients not to eat from the operated side, and to consume soft food during the study, advise patients not to force the suture thread with their tongue, and ensure the insertion point of the suture needle is at least 5 mm from the edge of the flap.

One of the advantages of absorbable suture threads is that generally there is no need to remove them. However, various tissue reactions have been reported as a result of the degradation of the absorbed suture thread by hydrolysis, enzymatic digestion, or phagocytosis. The rate of degradation depends on the pH and temperature of the tissues around the suture [15]. Also, intraoral sutures have unique problems compared to extraoral sutures, in speech or swallowing [11][17][18]. Therefore, it should be noted that the present study was a clinical study and it was not possible to control the pH or intraoral conditions of the patient such as muscle stretching, etc. These factors can create a gap between the edges of the wound and make the wound susceptible to infection due to the presence of saliva, food, and microorganisms.

## Conclusion

The results demonstrate that PGA sutures exhibit significantly higher tensile strength at all evaluated time points (baseline, 7 days, and 10 days post-surgery) compared to chromic catgut sutures. Specifically, the mean tensile strength of PGA sutures was 16.82 N at baseline, 14.56 N on day 7, and 11.50 N on day 10, whereas chromic catgut sutures had mean tensile strengths of 10.40 N, 8.76 N, and 6.45 N at the same respective time points. These findings suggest that PGA sutures are more durable and maintain their integrity better over the post-surgical period, which is crucial for optimal wound healing. The significant decrease in tensile strength over time for both suture types, particularly for chromic catgut, highlights the importance of selecting sutures that can provide sufficient support throughout the healing process. Surgeons should consider these results when choosing suture materials, especially in cases where higher tensile strength is needed to ensure effective wound closure and minimize the risk of complications.

## Conflict of Interest

None.
